# The Genetics of Brain Wiring: From Molecule to Mind

**DOI:** 10.1371/journal.pbio.0050113

**Published:** 2007-04-17

**Authors:** Kevin J Mitchell

## Abstract

What makes some people neurotic or schizophrenic or right-handed or fearless? The challenge in answering this is to map from genotype to anatomical and physiological phenotypes and beyond to behavior and cognition.

What makes some people neurotic or schizophrenic or right-handed or fearless? Are these behavioural differences caused by literal differences in how individuals' brains are wired? If so, what causes those differences? This age-old question of nature versus nurture can be recast in more realistic terms based on our modern understanding of genetics, development, and neuroscience. The challenge in this area is to understand how genotype is mapped to phenotype, not just in terms of the average effects of single genes across populations but also in terms of their combined effects in shaping the phenotypes of individuals.

*“Things are the way they are because they got that way.”—Gerald Weinberg*

There is compelling evidence that many psychiatric disorders have their origins in disturbed neurodevelopment, resulting in altered connectivity [1,2]. Similarly, many behavioural or cognitive traits are both heritable, at least moderately [3], and correlated with functional connectivity differences in various circuits [2]. The study of the genetics of behavioural or psychiatric traits may thus be directly informed by an understanding of the genetic architecture of the developmental processes underlying brain wiring. This essay presents a systems-level overview of these processes, highlighting several important properties that can have large effects on how genotype is mapped to phenotype: epistasis (meaning non-additive gene–gene interactions in this context), compensation, and stochastic developmental variation.

## Wiring the Brain

The establishment of the circuitry of the brain follows an intricate developmental programme involving cell fate specification, cell migration, axon pathfinding, target selection, and synaptogenesis [4]. The last four of these processes are mediated by small numbers of molecules in highly dynamic cellular substructures such as filopodia and dendritic spines [5]. As such, they are subject to a significant amount of noise at the biochemical level [6], because of fluctuations in the amounts of specific proteins, for example [7]. The complexity of the system as a whole results in buffering of this noise to give a reproducible developmental outcome [8]; in engineering terms the system is “robust” [9]. This robustness is due not only to molecular redundancy, but also to the involvement of multiple parallel pathways at each “choice point” (“degeneracy” [10,11]). Removal or alteration of many components individually may thus have little effect but will tend to sensitise the system to alterations in other components or to environmental stresses.

## No Gene Is an Island

These effects can be directly observed in model organisms. In Drosophila, the projection of motor axons to the embryonic body wall muscles has been very well characterised. In this system, many genes show no effect when removed on their own but do show an effect when ectopically expressed [12], a predicted characteristic of a robust system [9]. Many others show weak or incompletely penetrant phenotypes but strong interactions with other mutations both in molecularly related genes [13] and in unrelated genes operating in parallel pathways [14]. In some cases the direction of these interactions is both unpredictable and dependent on anatomical context [13].

Similar effects are seen for behavioural phenotypes, including the well-known large effects of genetic background in mice. Greenspan and colleagues discovered complex and shifting epistatic interactions between 16 genes for a behavioural trait in flies [15]. These findings reflect the dynamic nature of network interactions in a degenerate system; homeostatic mechanisms can often compensate for one insult by shifting weights in parallel pathways [11,16] (although these compensatory mechanisms may themselves be maladaptive in some contexts).

How this kind of epistasis observed at the biological level in model organisms relates to statistical epistasis in human populations is an open and critical question [17]. The importance of epistatic interactions is readily apparent in human studies, however, although difficult to measure using classical twin study designs. For example, the far greater concordance for autism of monozygotic compared to dizogytic twins (0.9 versus 0.1) [18] indicates that a large amount of the genetic variance underlying this disorder must be non-additive. Substantial non-additive genetic variance has also been observed and more precisely measured for personality traits in a combined twin and family study [19].

Researchers in psychiatric genetics are beginning to come to grips with the issue of epistasis. This will be especially important as data from large-scale whole-genome association studies emerge in the near future. Some of the methods of these studies look for epistasis between pairs of candidate genes, identified through suggestive single-gene associations with disease [20] or by involvement in the same signalling pathways [21]. These approaches are limited however to studying genes with significant effects on their own (which may be rare) or to looking for interactions only with genes in the same pathway (excluding unrelated genes, which we have seen above can have strong and unpredictable interactions). The use of model organisms to identify modifier genes (in an unbiased fashion) may provide an important third avenue to reveal candidate epistatic interactions. It may also be possible to mine whole-genome datasets in an unbiased fashion for all pairwise interactions. The view that this would lead to a quagmire of multiple testing corrections may be overly pessimistic; in one study, an exhaustive two-locus search in some scenarios detected combined effects even where each single locus had no significant effect alone. The increased power to detect effects outweighed the multiple corrections required, and held for a moderate but significant proportion of the space of possible allele frequencies, in which the majority of genetic variance is from the epistatic component [22]. How widely these conditions hold remains to be seen, but the development of these and similar strategies will be essential to interrogate the whole-genome association datasets currently being generated.

## The Winds of Chance

Another level of complexity arises in mapping from genotype to phenotype, even in the hypothetical case where whole-genome sequence is available and all single-gene and epistatic effects are known. Monozygotic twins in humans, and genetically identical organisms in other species, show considerable phenotypic variability. This phenotypic variability can be continuous or dichotomous and is observed for behavioural traits and psychiatric disorders [3] and also for anatomical phenotypes including neuronal connectivity [23]. This is true even in animal studies where the external environment and even the intra-uterine environment have been controlled as carefully as possible [24], suggesting an intrinsic source of variability. Such intrinsic variability is readily apparent in anatomical phenotypes in repeated structures within individual organisms, for example, on the two sides of the brain or in different segments of a Drosophila embryo [14].

Epigenetic differences have been proposed as a mechanism to explain this intrinsic phenotypic variability [25]. Random fluctuations in gene expression [7] at early stages of embryogenesis can be frozen in place through epigenetic chromatin modifications and clonally inherited in large numbers of cells. An analogous effect may arise through quite different mechanisms in the processes of brain wiring. There are a number of system-level properties that make brain wiring events very sensitive to small perturbations at certain time points. Threshold effects can emerge through the pioneering of tracts by small numbers of axons [26,27] or by non-neuronal cells [23,28], for example, and aberrant phenotypes can be set in place by the closure of critical periods, beyond which circuits are resistant to change [29]. Such threshold effects could lead to quite dichotomous outcomes in both anatomical phenotypes (normal versus absent corpus callosum [23], for example) and behavioural phenotypes (right-versus left-handed, or autistic or not).

**Figure 1:**
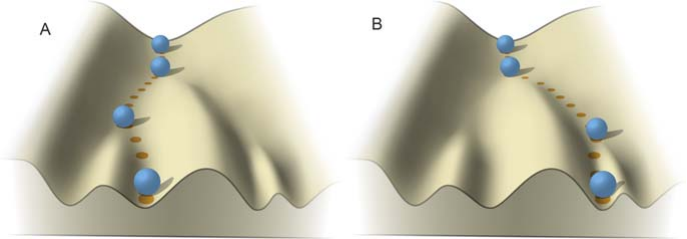
Waddington's “Epigenetic Landscape” These figures (adapted and redrawn from [30]) represent the indirect relationship of genotype to phenotype. An organism, represented by the ball, moves through development over a landscape with valleys representing various possible phenotypic states. The shape of this landscape is determined by an individual's overall genotype, which may have dramatic effects on the relative likelihood of different end points (for example, schizophrenic or not, or left- or right-handed). The diagrams represent two “runs” of the developmental process in two individuals (A and B) with the same starting genotype (as in monozygotic twins, for example). These two individuals therefore inherit the same probability of developing a certain phenotype but may have different actual phenotypic end points, determined by chance events and environmental effects, especially at critical points.

Waddington's “epigenetic landscape” (Figure 1) provides an elegant illustration of the nonlinear relationship between genotype and phenotype [30]. An organism, represented by a ball, moves through developmental time over an undulating landscape with a number of valleys representing potential phenotypic end points. The shape of this epigenetic landscape is determined by the organism's genotype, with the effects of individual genes (or combinations of genes) acting to increase or decrease the likelihood of passage into any particular valley. While these genotypic effects (importantly including sex effects) determine the probability of various phenotypes, the precise phenotype that actually emerges in an individual is also influenced by small random variation at any of a number of developmental “choice points” that can push an organism into a particular phenotypic valley, from which it becomes increasingly difficult to emerge. Environmental perturbations at these critical stages can have similar effects, depending on the underlying susceptibility to them (modelling gene × environment interactions).

Waddington referred to the buffering of developmental systems to produce a “wild-type” phenotype in the face of various mutations or environmental insults as “canalization” (though the definition of “wild-type” may be subjective—is right-handedness wild-type, for example?). Increasing mutational load should reduce the capacity for such buffering, leading to the prediction that individuals with greater developmental “noise” should be more susceptible to disease. Such noise can be indirectly measured by examining markers of “fluctuating asymmetry”, including fingerprint asymmetry, for example, which has indeed been found to be higher in individuals with schizophrenia [31].

## Putting It All Together

The challenge for the fields of neurodevelopmental and psychiatric genetics is to develop methods to take these factors into account in attempting to map from genotype to anatomical and physiological phenotypes and beyond to behaviour and cognition. Modelling this complexity may require both new mathematical methods and more detailed empirical data derived from studies of model organisms [32]. Whichever approaches are taken, it is clear that to understand the origins of individual differences in psychological traits we must keep developmental trajectories, and not just phenotypic end points, in mind.
